# Sustainable Dyeing of Nylon 6 Fabric with Turmeric (*Curcuma longa*) Extract Using an Infrared-Assisted Exhaust Process

**DOI:** 10.3390/molecules31213850

**Published:** 2026-10-25

**Authors:** Faiza Anwar, Imran Ahmad Khan, Asfandyar Khan, Kashif Javed, Nazmul Islam, Motahira Hashmi, Arsalan Ahmed, Fiaz Hussain

**Affiliations:** 1 Department of Textile and Apparel Science, School of Design and Textiles (SDT), University of Management & Technology, C-II Johar Town, Lahore 54770, Pakistan; faiza.anwar@umt.edu.pk (F.A.); imran.ahmad@umt.edu.pk (I.A.K.); asfandyar.khan@umt.edu.pk (A.K.); kashif.javed@umt.edu.pk (K.J.); 2 Department of Textile Engineering, Daffodil International University, Daffodil Smart City, Birulia, Dhaka 1216, Bangladesh; nazmul.te@daffodilvarsity.edu.bd; 3 Institute for Fiber Engineering and Science (IFES), Research Cluster for Social Implementation (RCSI), Shinshu University, Tokida, Ueda City 386-8567, Nagano, Japan; hashmi_motahira@shinshu-u.ac.jp; 4 Department of Textiles and Clothing, School of Engineering and Technology, National Textile University Karachi Campus, Karachi 74900, Pakistan; 5 Department of Mechanical Engineering, Gachon University, Seongnam-si 13120, Republic of Korea

**Keywords:** turmeric, nylon 6, IR-assisted dyeing, antioxidant activity, colorfastness, antibacterial activity

## Abstract

The present study focuses on the dyeing of knitted polyamide (nylon 6) fabric using turmeric (*Curcuma longa*), a natural dye source, through an infrared (IR)-assisted dyeing technique without the use of mordants and chemical auxiliaries. In this process, a 1% (*w*/*v*) aqueous turmeric dye extract was prepared and then applied on nylon 6 fabric through an IR-assisted exhaust dyeing method under different dyeing conditions, including variations in liquor ratio, dyeing time and temperature. The dyed samples were investigated for color strength (K/S), colorimetric properties (L*, a*, b*, ΔE*), surface morphology by scanning electron microscopy (SEM), elemental analysis by energy dispersive spectroscopy (EDS), antibacterial activities, antioxidant analysis and colorfastness (washing, light, and rubbing) properties according to standard testing protocols. The maximum K/S of 31.03 was observed under optimized conditions such as a liquor ratio of 1:30, a dyeing temperature of 110 °C, and a dyeing time of 60 min. The improved color strength was observed under optimized conditions such as a moderate temperature, a lower time and a high liquor ratio. SEM analysis revealed successful dyeing of nylon 6 without damaging the fiber surface morphology. The dyed samples showed good to excellent colorfastness properties, including rubbing (4–5), washing (4–5) and light fastness (5–6). The dyed fabrics also exhibited antibacterial activity against both Staphylococcus aureus (up to 87.46% reduction) and Escherichia coli (up to 79.08% reduction), and antioxidant activity of up to 75.15% in the 2,2-diphenyl-1-picrylhydrazyl (DPPH) radical scavenging assay. Thus, the IR-assisted dyeing process for nylon fabric is a promising dyeing method to dye synthetic textile fabric using natural colorant without the use of dyeing auxiliaries.

## 1. Introduction

Colors for clothing are normally selected on an esthetic basis with limited consideration given to their broader functional or physiological implications. However, the truth is that color has an impact on the environment [1]. Textile dyeing is one of the reasons for environmental pollution due to dyes containing effluents, hazardous auxiliaries and excessive water utilization [2]. Water pollution is the main environmental concern related to the dyeing process; it consumes significant amounts of water, toxic dyes and auxiliaries. The textile industry consumes approximately 0.6 gal/lb of water for woven fabrics and 111.8 gal/lb for felted textile goods [3]. This consumption of water depends upon multiple factors such as fabric type, dye type and processing equipment. Textile mills generate large volumes of wastewater due to various processing operations and hazardous chemical utilization [4]. During the application of dyes on different textile substrates, large amounts of dye effluents are generated due to improper dye uptake and dye fixation [5]. Textile mills discharge these effluents, which contain dyes and finishing chemicals such as sulfur, naphthol, vat dyes, nitrates, acetic acid, soaps, chromium compounds and heavy metals like copper, arsenic, lead, cadmium, mercury, nickel, and cobalt [6]. The other hazardous chemicals, such as dye fixers and softeners, are present in the form of formaldehyde and hydrocarbons [7]. Dyes are photolytically and chemically stable, making them difficult to degrade, leading to environmental hazards and subsequent bioaccumulation in the food web [8]. In recent years, EU regulations have compelled international trade markets to explore natural sources for dyeing of textile materials [9].

Natural dyes are an environmentally friendly way to color yarns and fabrics [10]. They add rich color to fabric that not only looks beautiful but also imparts functional properties to the textile materials. The other benefits related to natural dyes are that they have a far lower impact on the environment and health, as they are low in toxicity and biodegradability [11]. Natural dyes are eco-friendly, among their other best features. Natural dyes are derived from renewable resources, including grapes, fruits, and vegetables, as opposed to synthetic dyes, which are frequently manufactured from non-renewable materials [12].

Turmeric (*Curcuma longa*) is a natural dye extracted from the rhizomes of a plant which belongs to the Zingiberaceae family [13] which contains several bioactive compounds, primarily curcumin, curcuminoids, cinnamic acids, terpenoids, and other polyphenols [14]. Curcuminoids and their derivatives, including curcumin, demethoxycurcumin, and bisdemethoxycurcumin, carry significant antimicrobial, antioxidant and anti-inflammatory properties. It is used in the food and medical industries [15,16]. In the food industry, it used to increase the shelf life of products, while in the medical sector, it is used for pain relief, preventing cancer growth and improving blood circulation, and it possesses antibacterial, inflammatory, anti-tumor, and antioxidant properties [17]. The phenolic compound curcumin is used for the dyeing of textiles as a natural dye source due to its low toxicity and compatibility [18], and their dyeing properties are comparable to some reactive dyes. Previous studies have shown that the application of turmeric on natural fibers shows low wash fastness without mordant [19] and that it has low dye affinity towards cotton, leading to shade instability [20]. Bhatti et al. (2010) applied gamma radiation to enhance the color strength and colorfastness properties of cotton fabric using turmeric dyes [21].

Nylon fibers has played a very important role in the textile market and in other industries since its introduction in the 1930s. Nylon, being a synthetic fiber, has many versatile properties like strength and durability and is therefore widely used to produce lightweight, breathable, and strong fabrics [22,23]. Other than the textile industry, it is also used in carpets, upholstery, household products and the automotive industry for seat belts and airbags [24]. In conventional practices, nylon fabrics are normally dyed using synthetic dyes because they produce brighter colors, but these dyes have environmental concerns; in contrast, natural dyes obtained from renewable plants and animal sources are more sustainable and eco-friendly alternatives. For this reason, nylon has gained more popularity as a step toward more sustainable textile practices. Several studies have been conducted on the dyeing of nylon with natural colorants like berberine (*Berberis vulgaris*) [25], saffron (*Crocus sativus* L.), weld (*Reseda luteola* L.) [26], henna, cochineal [27], and turmeric [28], but a common issue was the use of metallic mordants, which cause heavy effluent loads and poor colorfastness ratings. Some researchers used bio-mordants to dye wool fibers with natural dyes [29] and established their dyeing characteristics using *Aesculus hippocastanum* extract [30].

Turmeric is known for its antioxidant, antimicrobial, and anti-inflammatory properties, which make it most suitable for textile applications [31]. Many studies have demonstrated the successful application of turmeric dyes on different textiles, including cotton, wool, and banana fibers [32], with satisfactory color strength and fastness results [14]. Furthermore, the utilization of bio-mordants has also been reported to increase dye fixation. Unlike synthetic dyes, the application of turmeric requires careful optimization of dyeing parameters, such as temperature and time. The infrared (IR) dyeing process is an alternative process to other advanced dyeing techniques used for better dyeing uptake and includes ultrasonic treatment [33], gamma radiation [34], plasma treatment [35], supercritical fluid extraction [36], microwave irradiation [37], and UV radiation. Researchers are applying various techniques to develop sustainable methods of dyeing with natural dyes. Eyupoglu et al. dyed plasma-treated silk fabric with *Hypericum Perforatum* and estimated the dyeing characteristics by using a neural network model [38].

To the best of our knowledge, IR-assisted exhaust dyeing of nylon 6 with turmeric dyestuff has not been reported. The simultaneous impact of the liquor ratio, temperature, and time on the color strength under IR heating and the fixation, antibacterial activity, and antioxidant activity of the mordant-free dyed nylon 6 has still not been investigated.

This study utilized turmeric as a natural dye source to dye a nylon 6 knitted fabric using IR without any auxiliaries such as mordant, wetting agent, sequestering, buffering or leveling agents. The dye liquor consisted only of dye, water and salt, and an infrared-assisted dyeing technique was used to produce higher shade depths by increasing dye mobility, leading to fast dye uptake and uniform dyeing results. The color strength (K/S), color coordinates (CIELAB), colorfastness, antibacterial (qualitative and quantitative) and antioxidant activities of the dyed nylon samples were established according to standard testing procedures.

## 2. Results and Discussions

Polyamide (Nylon 6) knitted fabric samples were dyed using an IR-assisted dyeing technique, and the samples were evaluated for K/S, CIELAB color coordinates, antibacterial activity, antioxidant activity and colorfastness properties. The obtained results are presented and discussed under the following separate headings.

### 2.1. Exhaustion and Fixation Assessment Results

**Figure 1:**
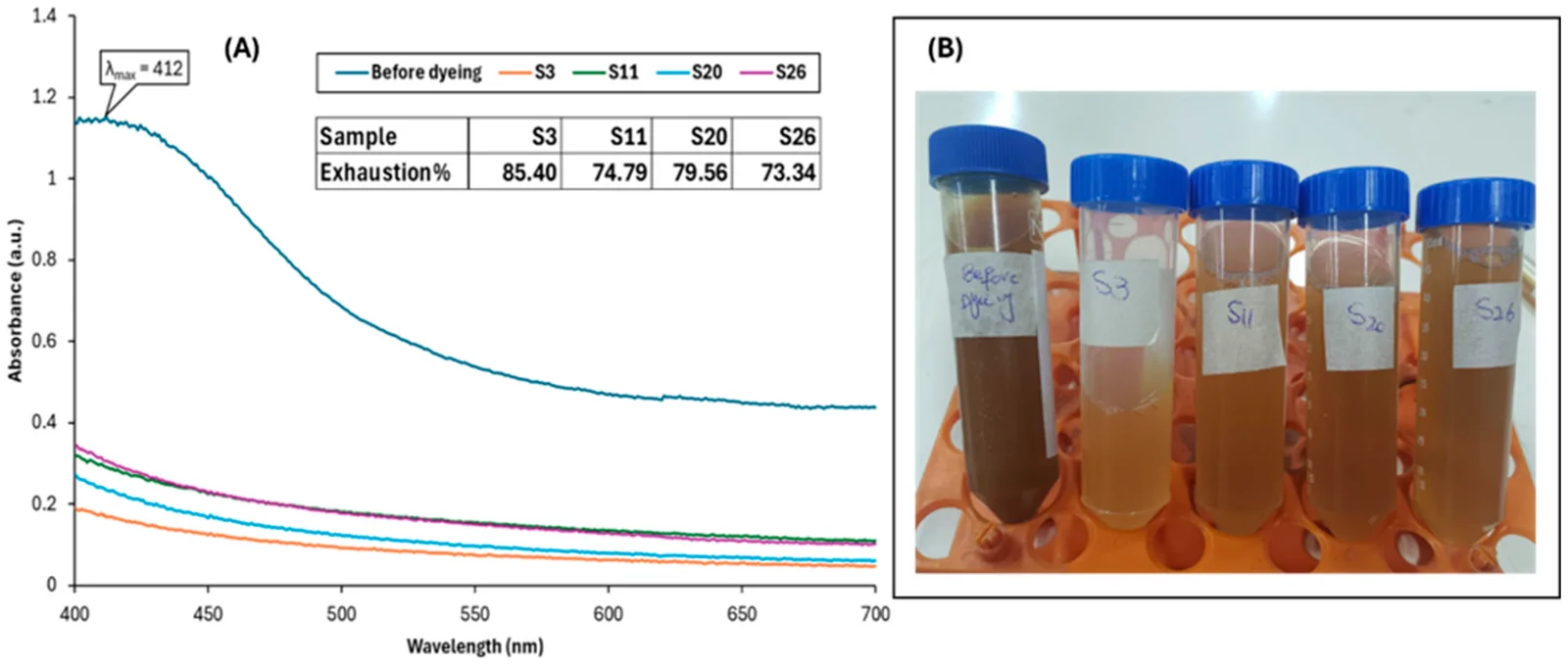
UV-visible spectra of the dye solutions before and after the dyeing process with exhaustion percentage values (**A**) and the visual appearance of the original solution before dyeing and after the dyeing processes for samples S3, S11, S20 and S26 (**B**).

The exhaustion percentages during the IR-dyeing of samples S3, S11, S20 and S26 were calculated using UV-visible spectroscopic analysis in the range of 400–700 nm (Figure 1A). It was observed that the dye solution before dyeing revealed λ_max_ at 412 nm, and then a smooth decreasing trend in the absorbance values continued until 700 nm. On the other hand, the dye solutions after the completion of the dyeing process (for samples S3, S11, S20 and S26) revealed a significant decrease in the absorbance values as compared to the solution before the dyeing process in the whole range of the spectrum, and the change in the dye solution can also be witnessed from the visual appearance in Figure 1B. The exhaustion percentage was the highest for sample S3 due to a lower liquor ratio (1:10), while samples S11, S20 and S26 revealed slightly lower exhaustion rates as compared to S3 due to the higher liquor ratios (1:20 for S11, and 1:30 for S20 and S26).

Subsequently, after the dyeing processes for samples S3, S11, S20 and S26, the fixation rates were calculated with the help of K/S values, directly after dyeing and then after soaping off (Table 1). It was observed that the fixation rate was 93% for sample S3, and interestingly, the exhaustion was higher for this sample. It is pertinent to mention that exhaustion and fixation are quite different phenomena, and it is very common in textile dyeing that the exhausted dye may not be completely fixed with the textile substrate. S11, S20 and S26 revealed higher fixation rates (98–99%) after rigorous washing off.

### 2.2. Color Strength and CIELAB Colorimetric Assessment

**Figure 2:**
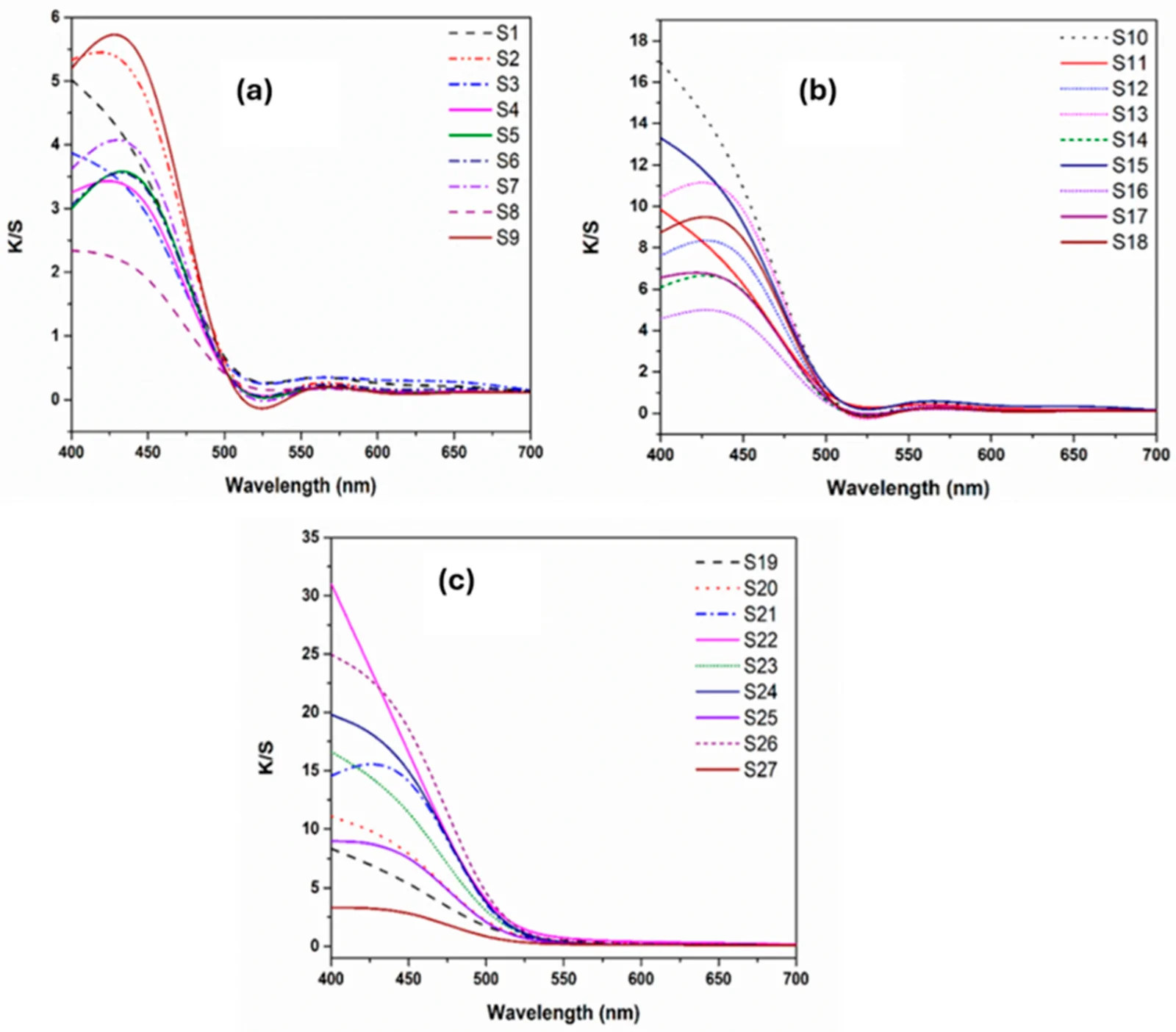
Color strength (K/S) variation in dyed nylon 6 fabrics as a consequence of changes in time and temperature while keeping the liquor ratio constant (**a**) 1:10, (**b**) 1:20, (**c**) 1:30.

Figure 2a illustrates the combined effects of time and temperature on the color strengths (K/S) of dyed samples while the liquor ratio was held constant at 1 10; the K/S values were measured in a wavelength range from 400 to 750 nm, while the λ_max_ for K/S values varied in the range of 400 to 450 nm. Among all the dyeing conditions, sample S2 exhibited the highest color strength (K/S = 5.33) at a temperature of 90 °C and a dyeing time of 120 min. The increase in shade depth is due to chain relaxation and improved diffusion of turmeric dye molecules into the fiber at that temperature [39]. In contrast, sample S8 exhibits lower color strength (K/S = 2.34) at the same temperature of 90 °C and a shorter dye time of 90 min, which indicates the importance of time; the low dye time causes low dye penetration, which changes the dye shade [39].

Similarly,Figure 2b depicts the combined effects of time and temperature on color strength of dyed samples while keeping the liquor ratio constant at 1:20. Sample S10 revealed a higher K/S value of 16.95 at a temperature of 110 °C and a time of 60 min, while the sample S16 (K/S = 4.57) revealed a lower shade depth at a dyeing temperature of 130 °C and a dyeing time of 90 min. In general, a temperature in the range near 130 °C is higher for nylon 6, which reduced the equilibrium absorption of dye molecules and ultimately reduced the shade depth [40].

A higher K/S value was observed for sample S22 (K/S = 31.03) at a liquor ratio of 1:30, a dyeing temperature of 110 °C and a dyeing time of 60 min, as illustrated in Figure 2c. This exceptionally high shade depth is due to the high liquor ratio allowing deep penetration of dye molecules into the amorphous regions of the fiber polymeric system [41]. The high color yield was also observed in the cases of S26 (K/S = 24.97) and S24 (K/S = 19.81), which revealed that a high liquor ratio, if supported by the optimum time and temperature, can increase the dye–fiber interaction, resulting in strong bonding for the development of durable textile materials. A lower shade depth was observed for S27 (K/S = 3.31); despite the high liquor ratio, the unfavorable combination of time and temperature hinders the dye penetration and lowered the color yield [42]. The overall results indicate a higher K/S value can be achieved at a higher liquor ratio combined with an optimum temperature around 110 °C and adequate time.

The CIELAB colorimetric analysis in Table 2 revealed noticeable changes in dyed nylon fabric as compared with the undyed sample where L* = 83.03, which shows white fabric. The decrease in (ΔL* = −4.06 to −18.55) in all dyed samples indicates successful dye deposition on nylon fabrics. A dark shade was observed for S21 (L* = 64.48) and S27 (L* = 65.54), corresponding to higher color strength. The a* values show a slight shift between green and reddish tones, but this shift minimally confirms that turmeric imparts yellow color more prominently. This is further supported by the increase in b* values (41.84–84.78); the highest yellowness level were observed for S17 (b* = 84.78), S5 (81.94), and S16 (81.10) due to curcumin. The overall (ΔEab) (41.95–83.50) values are high for all the samples, while S17 (83.5) and S5 (80.49) showed maximum values as compared to the untreated fabric. K/S values are not always directly correlated with *b* values; samples S22 (K/S = 31.03), followed by S26 (24.97), S24 (19.81), and S10 (16.95), showed high color strengths, showing better dye absorption. These samples generally exhibit moderate to low L* values and sufficiently high b*, confirming that deeper shades correspond to higher dye concentration within the fiber. In contrast, S8 (K/S = 2.34), S5 (2.99), S6 (3.05), and S27 (3.31) show lower color strength despite having moderate to high b* values, which indicates that yellowness does not mean higher dye uptake. So the overall results indicate that K/S values do not fully depend upon b* values, and they are inversely related to lightness (L*); ΔE*ab is affected by yellowness, and all these factors are affected by the dyeing conditions.

### 2.3. FTIR Analysis

**Figure 3:**
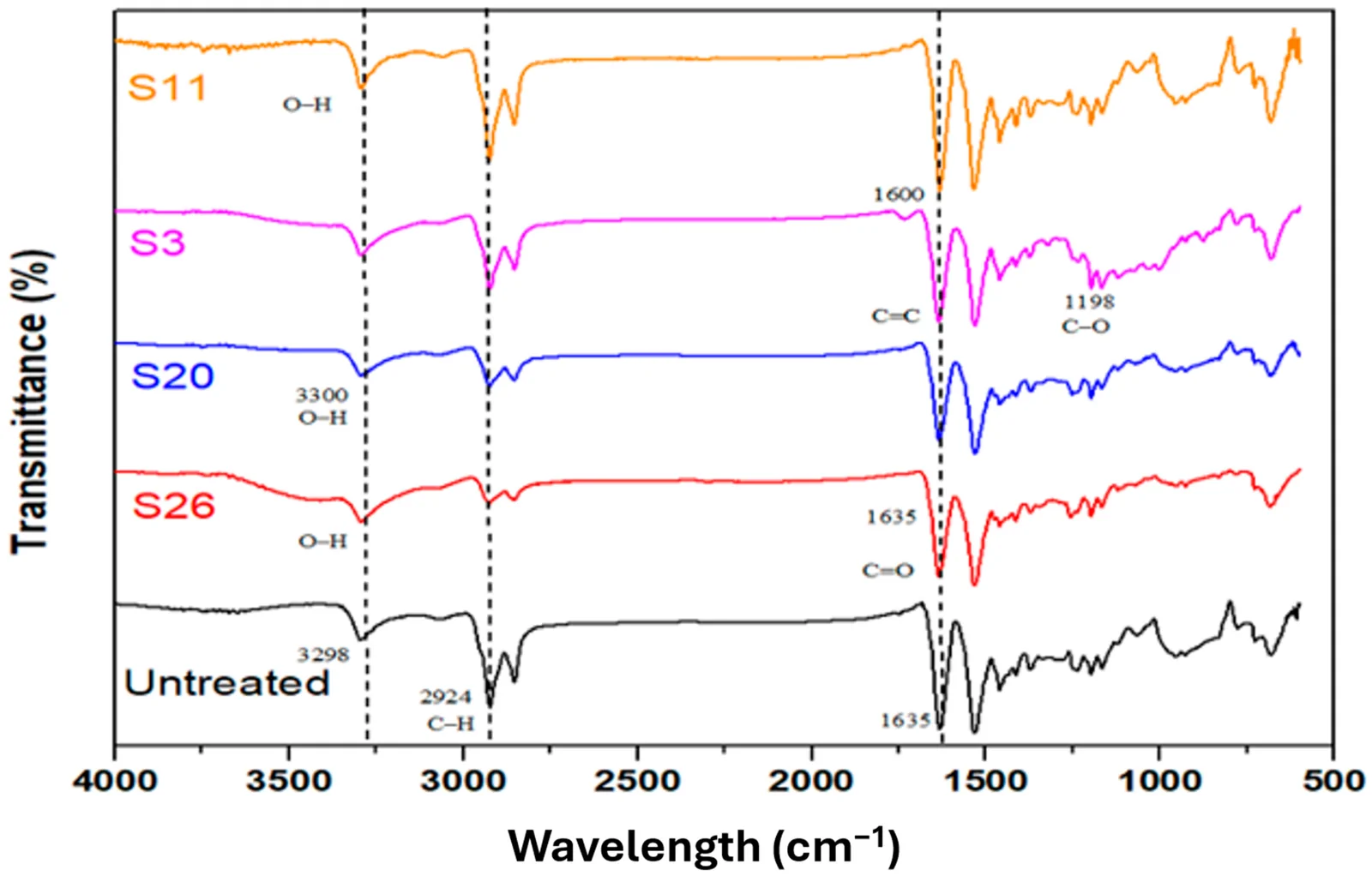
FTIR spectra of undyed and turmeric-extract-dyed nylon 6 samples following the IR-assisted dyeing technique.

FTIR is one of the most important characterization methods to identify the dominant functional groups in different materials. Selected samples (S3, S11, S20, S26) were analyzed by FTIR to explore the chemical modifications that occurred under different processing conditions. These samples were chosen for low, intermediate, and high liquor ratios and treatment conditions to compare the effect of the processing parameters on the chemical structure of the treated fabrics. Figure 3 shows the FTIR spectra of untreated nylon and dyed samples. The undyed nylon sample showed a major peak at 3298 cm^−1^ due to N–H stretching of amide groups [43], while another major peak was observed at 2924 cm^−1^ due to the C-H stretching of methylene groups in the polymer. A strong peak was seen at 1635 cm^−1^, which corresponds to C=O stretching [44]. However, the turmeric-dyed nylon sample S20 showed noticeable broadening at 3300 cm^−1^ due to hydroxyl (O-H) stretching from curcumin, the major coloring compound present in turmeric. Conversely, the amide group revealed a slight shift at 1635 cm^−1^. Samples S26 to S11 revealed a strong peak at 3200–3500 cm^−1^ due to the O-H stretching, which is the phenolic group of curcumin. As compared to the undyed nylon, this is a broad shift, which indicates hydrogen bonding between the OH group of curcumin and the amide groups (–CONH–) of nylon. The corresponding peaks at 1198 cm^−1^ is due to the C–O and C–N stretching. Overall, the FTIR spectra indicate the successful adsorption and coloration of turmeric dye on nylon fibers. The presence of aromatic C=C stretching, shifts in amide peaks, and broadening of O-H and N-H bands clearly indicate the dye–fiber interaction through hydrogen bonding. All the characteristic peak assignments and their corresponding functional groups are summarized in Table 3.

### 2.4. SEM Coupled with EDS Analysis

**Figure 4:**
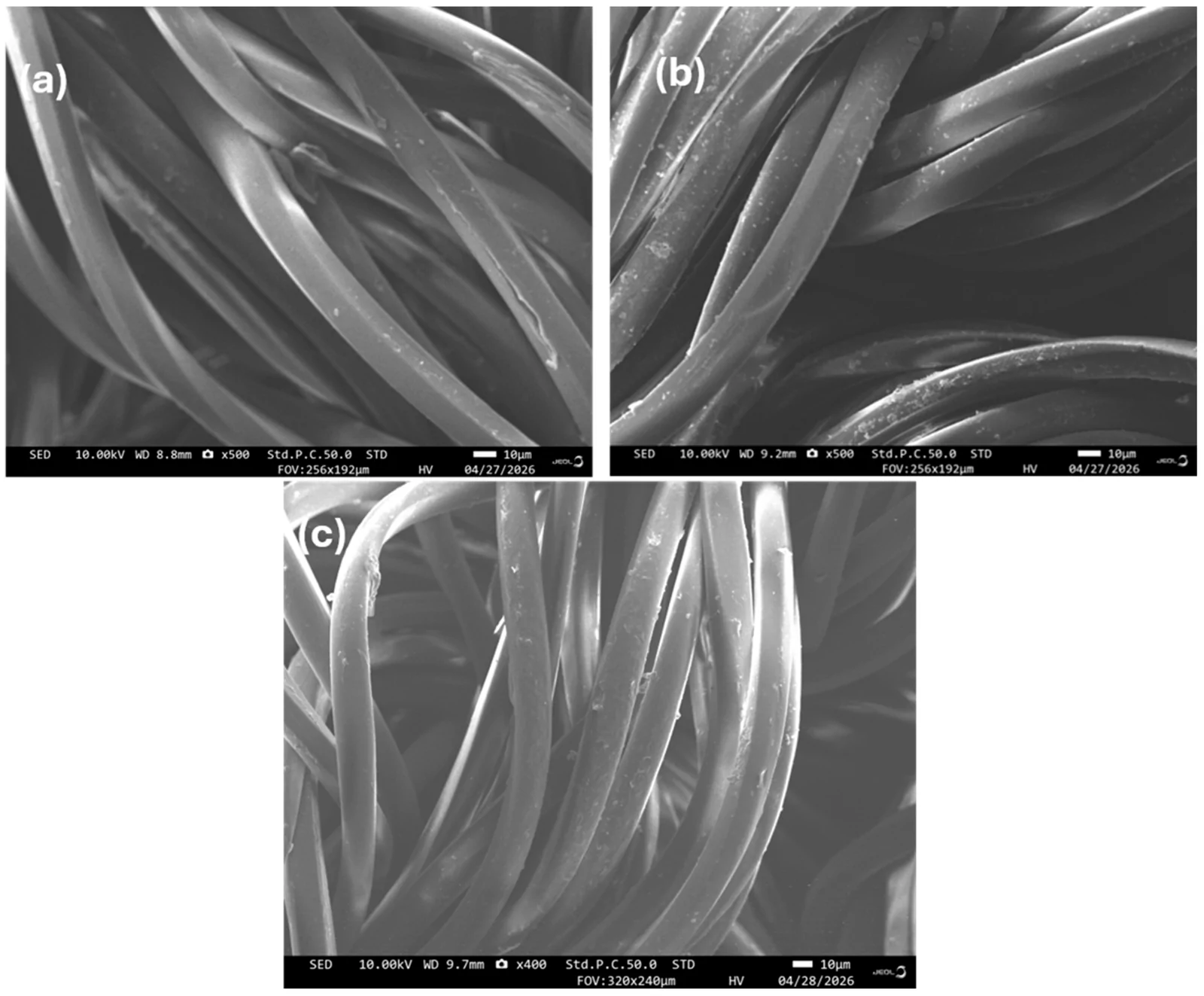
SEM analysis of undyed (**a**) and dyed nylon 6 samples S11 (**b**) and S20 (**c**) at a 10 µm resolution.

The surface morphology of dyed and undyed fabric nylon samples was examined by scanning electron microscopy. The SEM images of the undyed fabric (Figure 4a) and the dyed samples S11 and S20 (Figure 4b,c) were captured to observe structural changes in detail. The undyed samples showed a smooth surface, which is evidence for the absence of any surface attachments. In contrast, the dyed samples revealed the presence of some particulate matter on the surface of nylon fibers. There were no cracks or damage observed in the SEM images, which confirms that the fundamental surface of the nylon fiber remains preserved, with no evidence of fiber degradation after the IR-assisted dyeing process [31]. Thus, these findings showed that the turmeric extract served as an appropriate colorant to dye nylon in the IR-assisted dyeing process. The surface depositions come from the extract, which results in antibacterial and antioxidant properties of nylon fibers.

**Figure 5:**
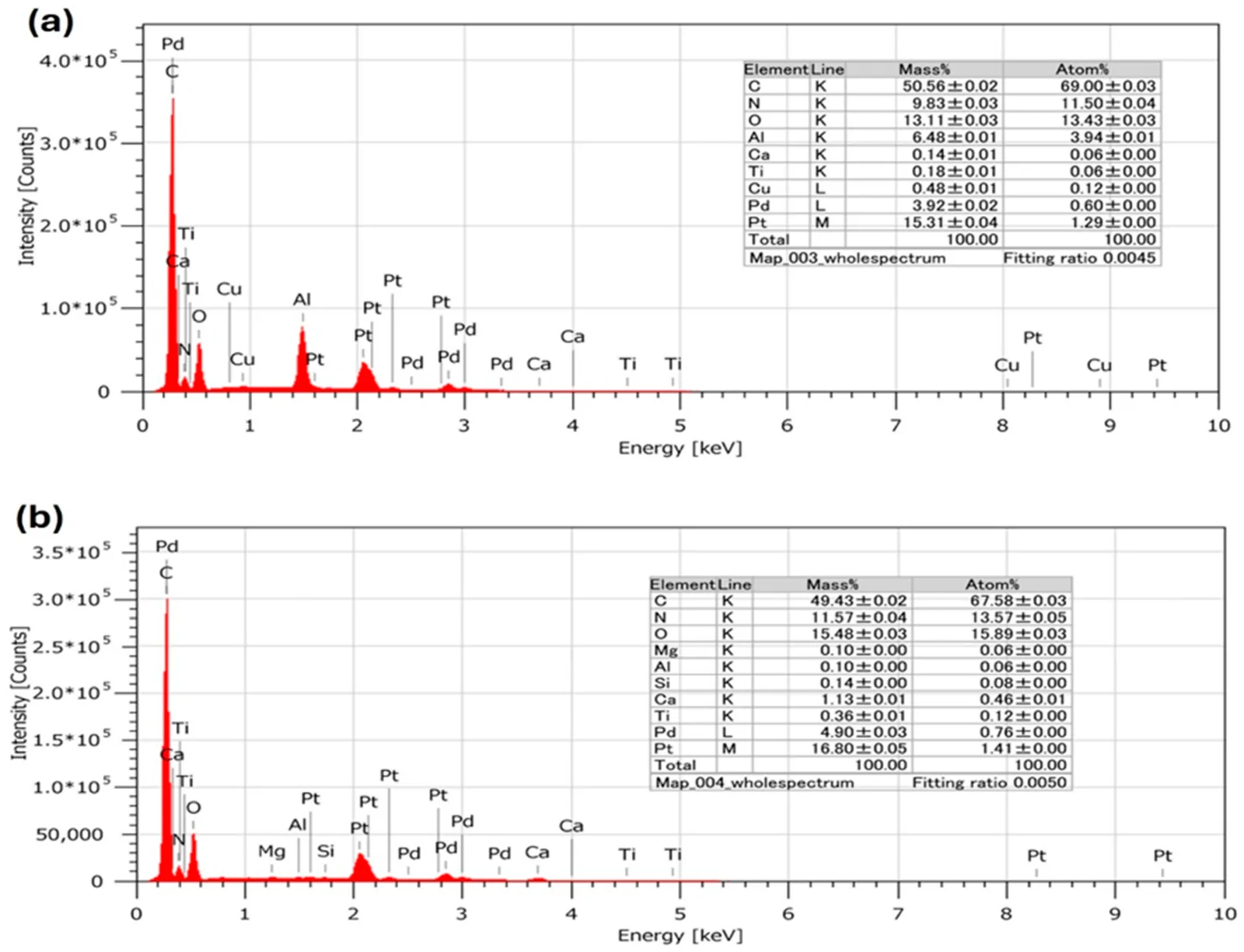
Energy-dispersive spectroscopic (EDS) spectra of the undyed (**a**) and dyed nylon 6 sample S11 (**b**).

Further confirmation was carried out using the typical EDS spectra of undyed (Figure 5a) and dyed nylon sample S11 (Figure 5b) with their respective elemental compositions. The spectra were characterized by the strong peaks of carbon, nitrogen and oxygen, which are the main components of nylon fiber. In contrast, the dyed samples revealed stronger peaks of oxygen, which indicate the successful deposition of turmeric dye on the fabric, including the phenolic group (curcumin structure) of turmeric, which contains oxygen and carbonyl groups. The comparison of these two spectra shows increases in oxygen content from 13.11% to 15.48% and nitrogen from 9.83% to 11.57%, which indicate the dye’s interaction with nylon through hydrogen bonding and suggest an increase in the functionalization potential. The increase in nitrogen content of the dyed fabric can be attributed to the presence of 1.20% nitrogen content in the turmeric rhizome.

### 2.5. Assessment of Antibacterial and Antioxidant Activities

**Figure 6:**
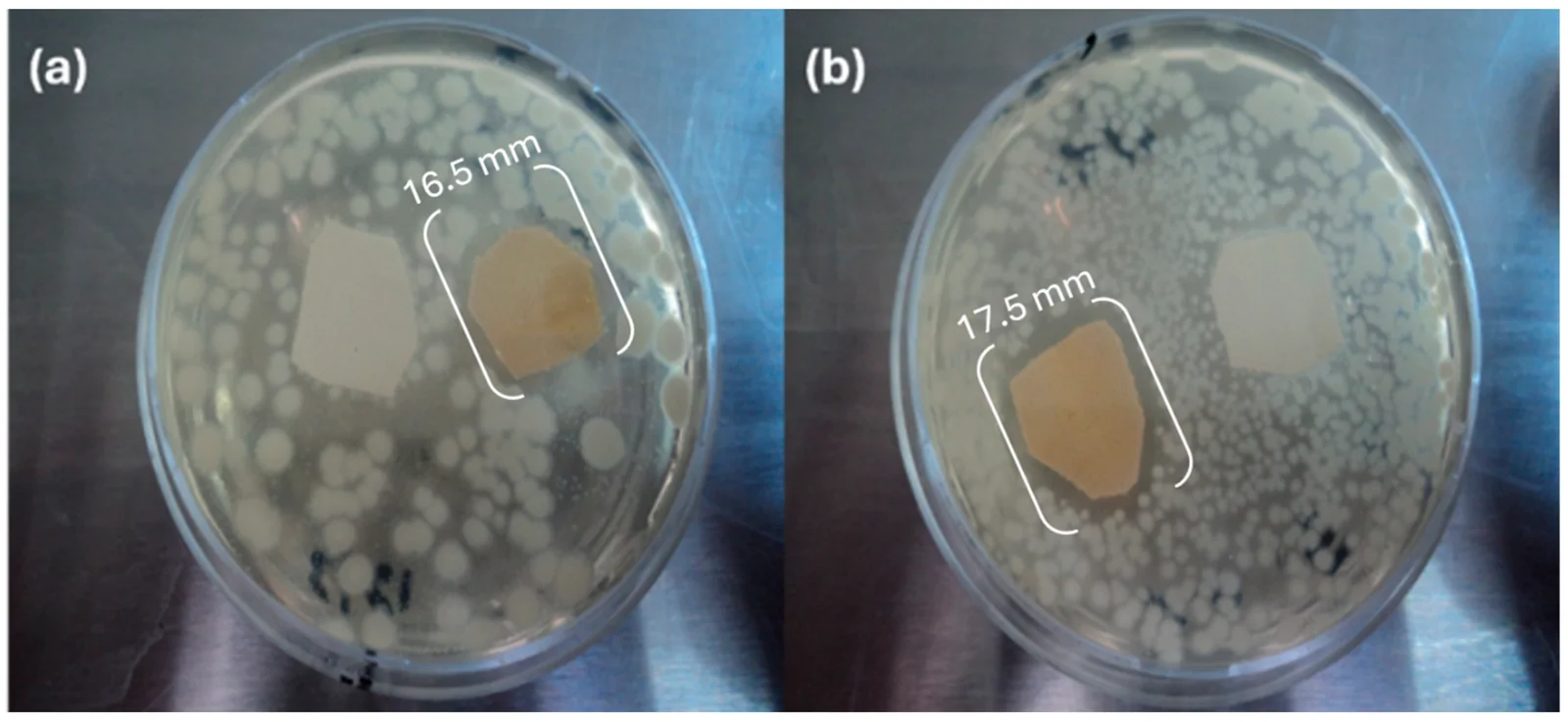
Antibacterial activities of the undyed and dyed sample (S20), showing the zone of inhibition against *Escherichia coli* (**a**) and *Staphylococcus aureus* (**b**) following the qualitative antibacterial testing protocol (AATCC TM 147).

The antibacterial properties of dyed and undyed samples against *Escherichia coli* (*E. coli* ATCC 25922) and *Staphylococcus aureus* (*S. aureus* ATCC 25923), with dyeing performed according to the qualitative standard test method (AATCC TM 147), are shown in Figure 6a and Figure 6b, respectively. The zone of inhibition was clearer and larger in diameter (17.5 mm) in the of the Gram-positive bacterial strain (*S. aureus*), while in the case of the Gram-negative strain (*E. coli*), the zone of inhibition was smaller (16.5 mm); this is because the Gram-positive bacterial strain hass a simpler cell wall, making it easy for curcumin to act, while Gram-negative bacteria have an outer membrane that acts as a barrie, makes it difficult for curcumin to diffuse, and reduces the effectiveness of antibacterial activity [45,46]. In the case of undyed samples, the zone of inhibition was absent, showing the growth of bacteria on the surface of the samples as well.

The antibacterial results were further confirmed by following AATCC TM 100 (quantitatively) using the same bacterial strains. Undyed control sample (nylon) and dyed samples S3, S11, S20 and S26 were examined (Table 4). The colony-forming units (CFUs) were calculated at the start of the experiment (t_o_) and at time 24 h (t_1_). The control sample revealed no significant reduction in bacterial growth using the *S. aureus* strain, while the dyed samples (S3, S11, S20 and S26) revealed above 80% reduction in bacterial CFUs after 24 h incubation time. Similarly, in the antibacterial assessment using *E. coli*, the dyed samples showed less reduction in CFUs after 24 h incubation as compared to *S. aureus.* The results are in agreement with the qualitative antibacterial assessment using AATCC TM 147, which can be attributed to the stronger outer membrane of Gram-negative bacterium.

**Figure 7:**
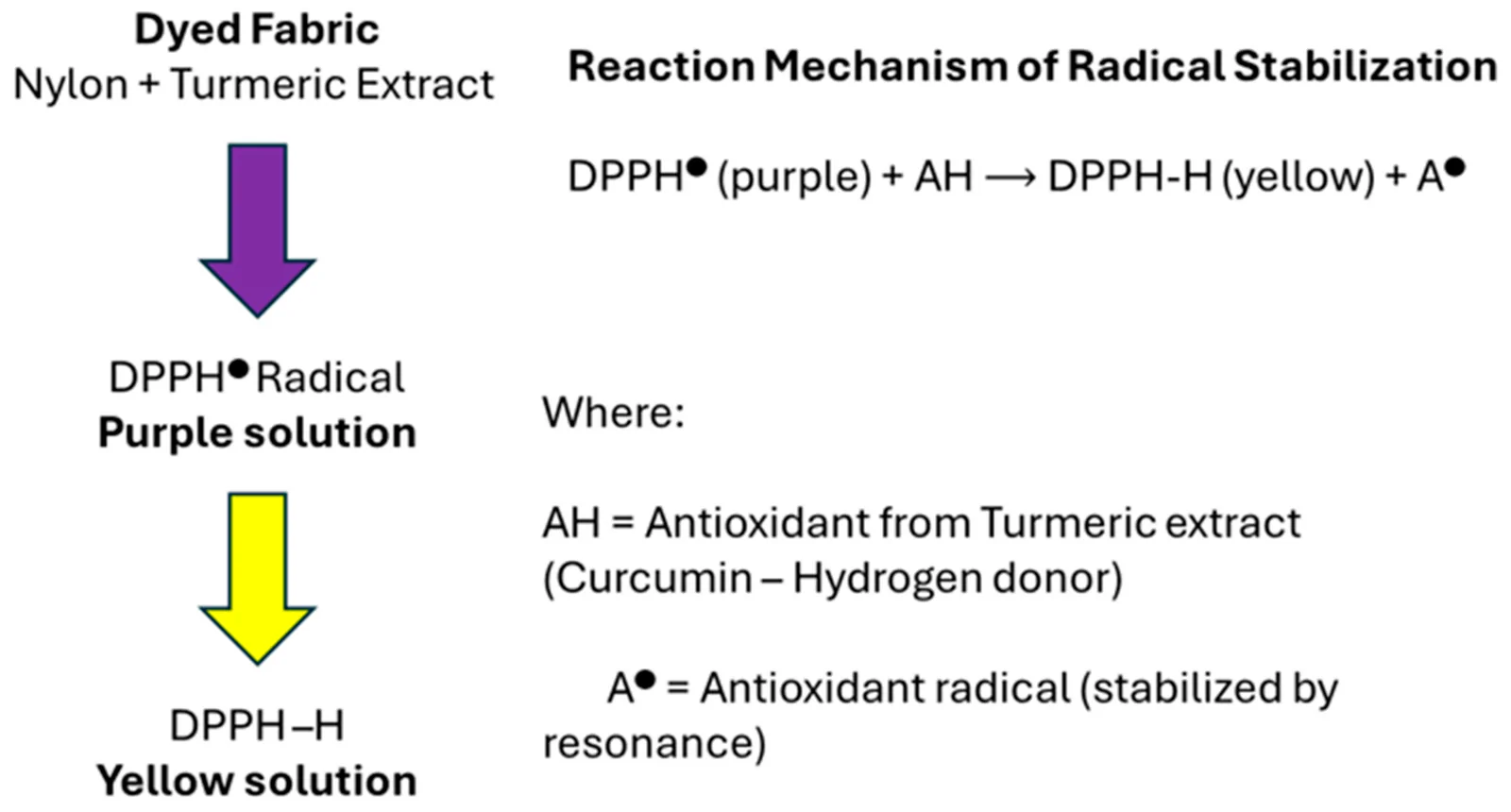
Proposed mechanism of DPPH^●^ conversion into DPPH-H by the action of turmeric extract (curcumin).

The antioxidant activity of the undyed and dyed nylon samples was established in an in vitro antioxidant assay. The undyed control sample and dyed samples S3, S11, S20 and S26 were examined, and the % antioxidant activities were calculated using the absorbance of the DPPH^●^ solution after 30 min of the incubation process (Table 5). The undyed nylon (control sample) revealed no significant color changes in the DPPH^●^ solution before and after incubation, and thus the activity against oxidation was negligible (5.64%). Interestingly, the nylon sample (S3) dyed with turmeric extract showed 68.10% antioxidant activity, while the highest activity (75.15%) was observed in the case of S11. The activities of samples S20 and S26 were observed to be 71.56 and 70.25%, respectively. The turmeric extract contains several antioxidant compounds, and curcumin is one of the most prominent, which has proven its potential in the traditional treatments of incurable diseases due to its antioxidant activity [47,48]. The stabilization of DPPH^●^ into DPPH-H can be understood from the detailed mechanism shown in Figure 7. Several research works have established the antioxidant, antibacterial, and antifungal activities of turmeric extract. Thus, this research work confirms its application as a dye for nylon and its function properties [49].

### 2.6. Colorfastness Assessments Results

**Figure 8:**
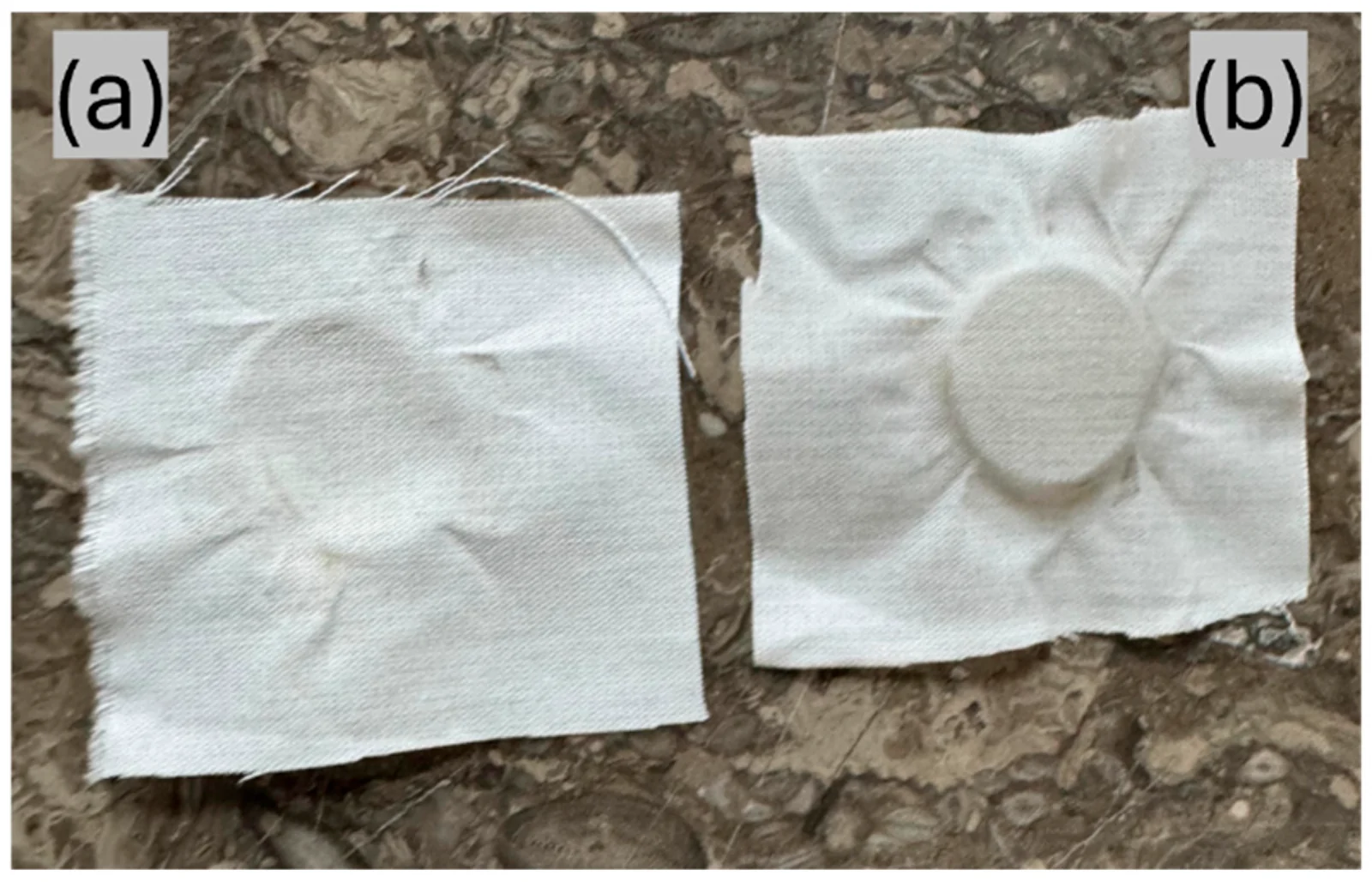
Visual assessment of dry rubbing fastness (**a**) and wet rubbing fastness (**b**) of the dyed nylon 6 samples following the standard protocol (ISO 105-X12).

**Figure 9:**
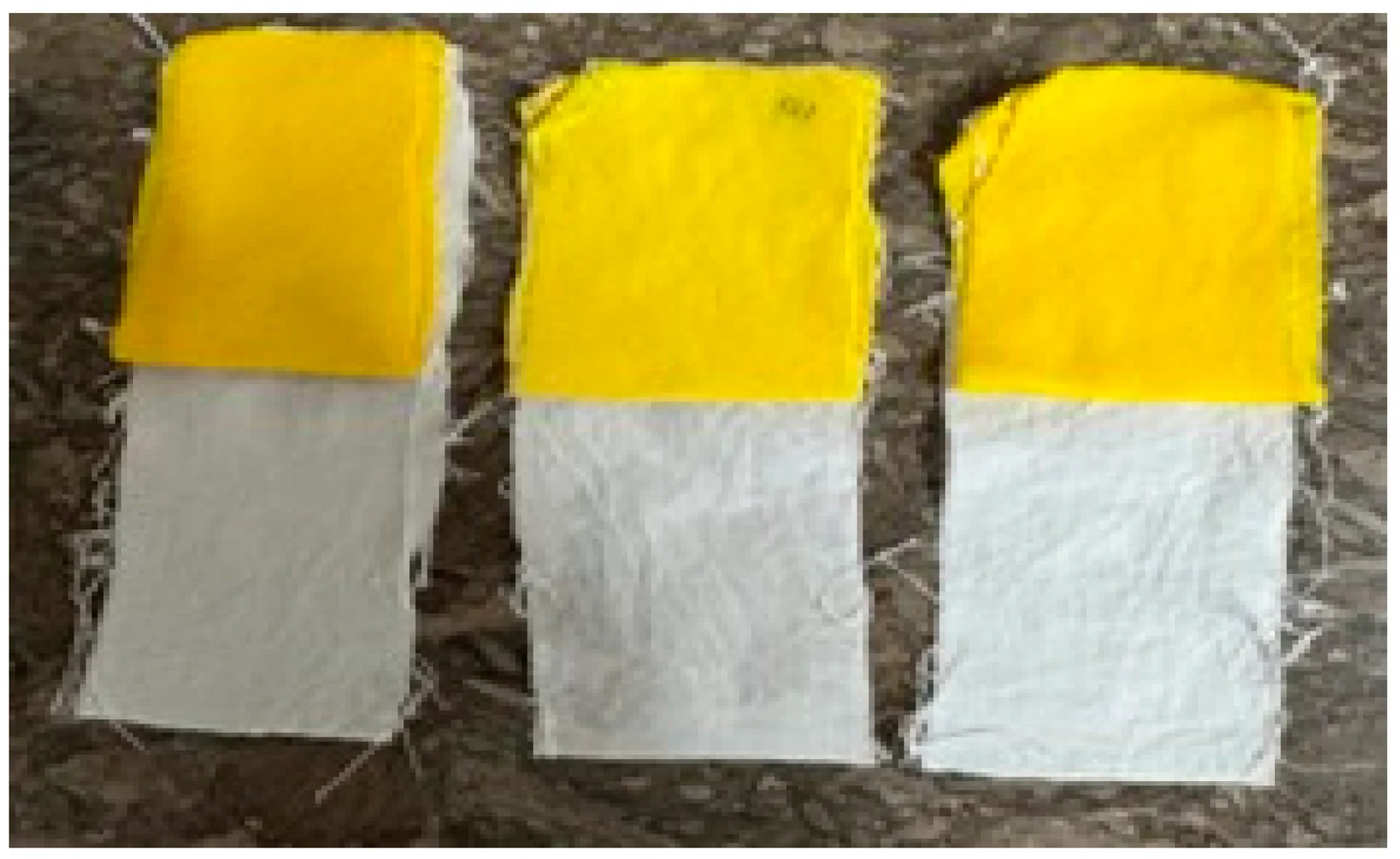
Visual assessment of washing fastness results of IR-assisted dyed nylon 6 fabric with a cotton-adjacent strip through ISO 105-C06.

The colorfastness results of IR-assisted dyeing of nylon knitted fabric with different combinations of dyeing temperature and dyeing time are shown in Table 6. In the case of rubbing fastness, all the samples show a rating of 4–5 (excellent) in both dry rubbing and wet rubbing, and the visual confirmation revealed no staining on bleached cotton, as can be seen in Figure 8. The gray scale values range from 1 to 5, where 1 indicates poor fastness while 5 indicates excellent fastness properties. In the case of washing fastness, the results are remarkably consistent at 4–5 (excellent). Figure 9 shows evidence of no staining on bleached cotton strips attached to the dyed nylon during the washing fastness protocol. This excellent fastness is because of fact that unlike the conventional dyeing process, IR radiation penetrates directly into the fabric and excites the chemical structure of the dye and fabric, resulting in more uniform dye diffusion. This enhanced diffusion caused the curcumin compound in the turmeric to penetrate the amorphous regions of fabric [50,51]. The light fastness ratings of the dyed nylon samples were recorded according to the blue wool scale, and the values were very similar in terms of ratings 5–6 (very good). The relatively lower light fastness can be attributed to the fact that the natural dyes’ chromophores are prone to degradation due to prolonged exposure to UV radiation.

### 2.7. Statistical Analysis of K/S Values

The color strength (K/S) of the treated fabrics was statistically assessed by three-way factorial analysis of variance (ANOVA) with the liquor ratio, treatment temperature, and treatment time as factors. The results of ANOVA (Table 7) showed that all three processing parameters significantly influenced the K/S value at a *p*-value < 0.001. Liquor ratio (LR) had the most significant effect on color strength (F = 835.497) among the investigated factors, indicating that the liquor-to-fabric ratio was the most significant parameter that influences the dye uptake and color development. The K/S values were also highly dependent (statistically significant) on temperature (F = 129.418) and treatment duration (F = 44.029). These results have shown that all the processing parameters have their own unique contribution to dyeing performance and color strength.

Furthermore, the main effects of most of the interaction terms were significant at a very high level (*p* < 0.001). The liquor ratio x temperature interaction (F = 117.971) showed that the effect of the treatment temperature on the color strength varied according to the liquor ratio chosen. Similarly, liquor ratio × time (F = 24.722) and temperature × time (F = 143.024) interactions were both significant. Moreover, the interactions between liquor ratio, temperature, and treatment time are also statistically significant (F = 82.698), which means that the combined effect of these three processing variables affects the final color strength. The results show that there are strong interactions between the investigated parameters, which indicate that they act not independently of each other but synergistically, and the combination of these parameters controls the dyeing performance.

Overall, the three-way factorial ANOVA gives strong statistical results that are in accordance with the experimental observations and validate the optimized dyeing conditions described in this study. The significant interaction effects also confirm that optimizing one processing variable at a time is not an effective approach to predict the dyeing performance, and thus the liquor ratio, treatment temperature, and treatment time should be considered together to attain better color strength and better process repeatability.

## 3. Materials and Methods

### 3.1. Materials

A commercially scoured and bleached single jersey nylon 6 knitted fabric with an areal density of 170 g/m^2^, 55 courses per inch (CPI), and 40 wales per inch (WPI) was purchased from Master Textile Mills (Pvt.) Ltd. Faisalabad, Pakistan, and it was used as a substrate throughout the study. Turmeric powder (food-grade) was obtained from Township Market, Lahore, Pakistan. *Escherichia coli* (Gram-negative bacterial strain) and *Staphylococcus aureus* (Gram-positive bacterial strain) were used as test organisms and are common causes of certain infections. *Escherichia coli* (*E. coli* ATCC 25922) and *Staphylococcus aureus* (*S. aureus* ATCC 25923) strains were obtained from the University of Veterinary and Animal Sciences (UVAS), Lahore, Pakistan. Sodium chloride (≥97%purity) was purchased from Sigma-Aldrich (St. Louis, MO, USA). All chemicals were used as received without further purification.

### 3.2. Extraction and Application

First, 1% (*w*/*v*) dye stock solution was prepared by adding 10 g of turmeric dye powder to 1000 mL of distilled water. The resultant mixture was then boiled for 3 h at 100 °C. After boiling, the mixture was cooled down and filtered through voile fabric. The obtained filtrate was used for dyeing of nylon fabric according to the designed design of the experiment. The yellow-colored filtrate contains curcuminoids and their derivatives, which are responsible for its yellow color and functional properties.

**Figure 10:**
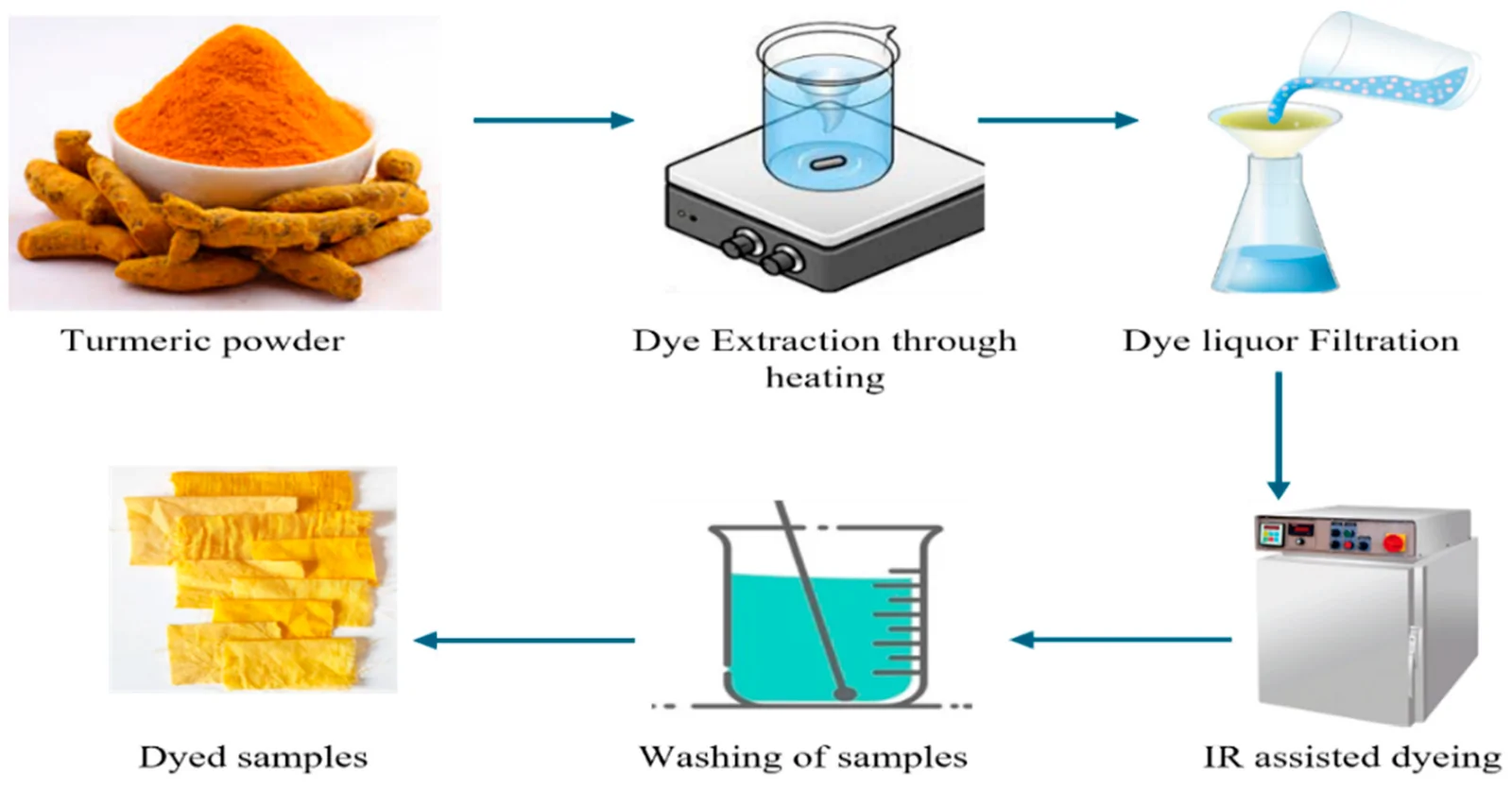
Schematic representation of IR-assisted dyeing of nylon using turmeric extract via the exhaust method.

The nylon 6 knitted fabric was dyed using a lab-scale infrared dyeing machine (Exact Dye L15Pro) Dongguan, China, as shown in Figure 10.

The dyeing parameters were maintained according to the DOE, and a total of 27 samples were developed from S1–S27, as shown in Table 8. Three dyeing liquor ratios (1:10, 1:20, and 1:30), three dyeing temperatures (90, 110, and 130 °C) and three dyeing times (60, 90 and 120 min) were investigated to achieve optimum dyeing conditions. For instance, sodium chloride concentration was 5 g/L, which was kept constant through all experiments. After successful dyeing, the fabrics were washed with non-ionic standard soap at 2 g/L and 60 °C for 15 min. Samples were dried after rinsing with clean water. The dried samples were investigated for K/S, CIELAB, colorfastness, antibacterial and antioxidant activities.

### 3.3. Testing Methods

#### 3.3.1. Dye Exhaustion and Fixation Assessment

The exhaustion values were calculated by spectrophotometrically measuring the absorbance of the dye solutions before and after dyeing nylon samples (S3, S11, S20 and S26) with turmeric extracts using the following Equation (1).

(1) $$Exhaustion\;\%=\frac{A_{1}-A_{2}}{A_{1}}\times 100$$

In Equation (1), A_1_ is the absorbance of dye solution at λ_max_ before dyeing and A_2_ is the absorbance of the dye solution at λ_max_ after the completion of dyeing.

For the fixation of dyes on nylon, the color strength of the washed-off fabric (K/S)_m_ to that of the unwashed (or soap-treated) fabric (K/S)_o_ using the ratio given in the following Equation (2) was applied and the percentage values were recorded.

(2) $$Fixation\;rate=\frac{{\left(K/S\right)}_{m}}{{\left(K/S\right)}_{o}}\times 100$$

#### 3.3.2. Color Strength (K/S) and Color Coordinates CIELAB Assessments

The colorimetric characteristics of turmeric-dyed knitted nylon samples were obtained through a Spectra Flash-Data Color spectrophotometer (SF-600, Suzhou, China). The color yield (K/S) and CIELAB values were calculated using the Kubelka–Munk Equation (3). In this equation, K is the absorption, S is the scattering coefficient, and R is the reflectance of the turmeric-dyed sample (expressed as a decimal) measured at the wavelength where absorption is highest (maximum wavelength).

(3) $$\frac{K}{S}=\frac{{\left(1-R\right)}^{2}}{2R}$$

CIELAB represents the color in terms of L*, a*, b* and ΔE*ab, where L* represents the lightness and darkness of samples, ranging from 0 to 100, where 0 represents black and 100 is white; positive a* represents redness and negative a* represent greenness of samples. Positive b* indicates yellowness and negative b* blueness of an object, ΔE*ab represents how two colors are different from each other. A low value means colors are very similar, while a high value shows a noticeable difference in the obtained colors.

#### 3.3.3. FTIR Analysis Method

The FTIR analysis of dyed samples was measured by using FTIR-4600 Series—by JASCO (Tokyo, Japan)—in the wavelength range of 4000–400 cm^−1^ at room temperature. The spectra of dyed and undyed samples were analyzed by comparing the characteristic peaks to find out the dominant functional groups in the undyed and dyed nylon fabrics.

#### 3.3.4. SEM/EDS

The surface morphology of turmeric-dyed and undyed nylon fabrics was studied using a field-emission scanning electron microscope FE-SEM, Model: TESCAN MIRA3MU (Brno, Czech Republic). To investigate the elemental composition and their respective weight percentages, energy-dispersive X-ray spectroscopy (EDS) was applied. SEM images were recorded at 10 kV, and samples were gold-plated before examination.

#### 3.3.5. Antibacterial and Antioxidant Assessments Methods

The agar diffusion method was used to find out the antibacterial activity of both dyed and undyed samples by following the qualitative antibacterial protocol (AATCC TM 147) [52]. By following the protocol, nutrient agar was prepared by sterilizing it at 121 °C for 15 min, and the prepared agar solution was poured into sterilized Petri dishes and allowed to solidify. The test strains *S. aureus* and *E. coli* were cultured in nutrient broth at 37 °C for 24 h. After that, the turbidity of the suspension was adjusted to around 10^5^–10^6^ CFU/mL. The resultant bacterial suspension (0.1 mL) was spread on agar plates equally. The dyed and undyed sterilized samples were cut into standard size and placed on the inoculated agar surface, and plates were incubated for 37 °C for 18 h. After completion of the incubation time, samples were assessed by observing the zone of inhibition around the samples [53]. Besides the zone of inhibition, the bacterial growth on the surface of the samples was also investigated and recorded. In order to validate the qualitative antibacterial results, the standard quantitative test (AATCC TM 100) method was followed using the same bacterial strains. The reduction in colony-forming units (CFUs) was measured, and percentage reduction values were established.

The dyed nylon samples were examined for antioxidant activity (free-radical scavenging ability) according to the standard in vitro antioxidant assay [54,55]. To perform this test, a DPPH (2,2-diphenyl-1-picrylhydrazyl) radical was mixed in a 0.15 mM methanol solution. DPPH contains two dominant peaks, one in the UV region in the range of 320–330 nm and another in the visible region at 517–518 nm, showing a deep purple color. The dyed and undyed nylon samples were immersed in the DPPH radical methanol solution and incubated at 37 °C for 30 min in a dark environment. During the incubation period, the antioxidants present on the dyed nylon react with the DPPH radical, which can be witnessed by the change in the color of the DPPH radical methanol solution from deep purple to yellow as a consequence of the reduction process of DPPH. In the case of no antioxidant activity, the DPPH methanol solution’s color remains the same as it is at the start of incubation. The antioxidant activity (%) was calculated by following Equation (4) as follows:

(4) $$Antioxidant\;activity\left(\%\right)=\frac{A_{c}-A_{s}}{A_{c}}\times 100$$

In Equation (4), A_c_ stands for the absorbance of the DPPH methanol solution containing the undyed nylon sample, while A_s_ stands for the absorbance of the DPPH methanol solution containing the dyed nylon sample. The obtained percent value demonstrates the antioxidant activity, where a value higher than 80% shows strong reduction potential of the present antioxidants on the developed nylon fabrics.

#### 3.3.6. Colorfastness Assessments

The colorfastness to rubbing, washing and lighting of dyed nylon 6 samples was examined to establish the durability of the developed textile material [56,57]. The washing fastness test was carried out in such a way that the test specimen was first stitched with cotton fabric (standard) for staining according to (ISO 105-C06). A temperature of 40 °C was used for 30 min in a Launder-O-meter using the ECE reference detergent. The shade changes and staining on cotton fabric were evaluated using a gray scale (1–5). The dry and wet rubbing fastness of the dyed samples were measured through a lab-scale crock meter following the ISO 105-X12 standard testing protocol. The staining on bleached cotton fabric during the rubbing fastness protocol was recorded using gray scale (1–5). The colorfastness to light of the dyed samples was measured according to (ISO 105-B02) by exposing the samples to artificial light using a xenon arc lamp under controlled conditions, and the ratings were determined using the blue wool scale (1–8). The fastness was assessed visually by two trained assessors in separate tests under standard illumination (D65). Three replicate specimens were tested for each sample and each test. The average of the assessors’ ratings was reported to the nearest half grade.

## 4. Conclusions

In the present research, turmeric dye was successfully applied to nylon fabric using infrared (IR)-assisted dyeing without the use of any hazardous chemicals or mordants. Findings revealed that IR dyeing is an environmentally friendly approach for increasing dye uptake and uniform dye application on polyamide fibers. Color strength and colorimetric values are significantly affected by dyeing conditions like temperature, time and liquor ratio. Among these, temperatures around 110 °C, in combination with adequate time and a high liquor ratio, can increase the color strength, while in contrast, a high temperature of 130 °C adversely affects the dye uptake because the high temperature reduce the dye absorption due to the structural limitations of nylon. Colorimetric analysis indicated a significant decrease in lightness (L*) and a marked increase in yellowness (b*) values, confirming effective coloration imparted by curcumin. The functional characteristics of dyed samples were also promising, as dyed samples show antibacterial activity against both Gram-positive (*S. aureus*) and Gram-negative (*E. coli*) bacteria. The antioxidant properties of the S3, S11, S20 and S26 were also good, which further confirms the functionality of the developed fabrics. The fastness properties were good to excellent, indicating proper dye fixation under an IR heating system.

Overall, this research establishes that IR-assisted dyeing with turmeric is a viable method for nylon 6 fabrics, which imparts good color strength, color fastness and functional properties.

## 5. Limitations and Future Recommendations

This research explored the application of turmeric extracts on nylon 6 via the IR-assisted exhaust dyeing method. In general, natural dyeing is sustainable and provides functional properties to the substrates, but several major challenges still exist in commercial scalability. Some limitations of this study are given below, which are recommended for further exploration.

I. The possible turmeric dye diffusion mechanism under IR-assisted heating and the effect of temperature on nylon chain mobility need further investigation.
II. The determination of chemical interaction models, adsorption mechanism, thermodynamic analysis and bonding energy evaluation should be explored by experts on pure chemistry.
III. The potential effects of IR heating on the physical and mechanical properties of nylon 6 should be established.
IV. Food-grade turmeric powder was used, where the content of curcuminoids had not been quantified (e.g., HPLC). Thus, it can be concluded from the evidence of UV–Vis and the literature that the color and functional properties are attributed to curcuminoids. In the future, HPLC quantification of the extract is suggested.

## Figures and Tables

**Table 1:** Determination of fixation rates (from K/S values) of turmeric dyes before and after washing off for nylon samples S3, S11, S20 and S26.

Sample Name	K/S Before Wash	K/S After Wash	Fixation Rate %
S3	3.87	3.70	95
S11	9.85	9.78	99
S20	11.09	10.91	98
S26	24.97	24.93	99

**Table 2:** CIELAB color coordinates assessment and comparison of the IR-assisted exhaust dyed nylon 6 fabrics using turmeric extract.

Serial Number	L*	a*	b*	ΔL*	Δa*	Δb*	ΔE*ab	K/S	Dyed Sample
Untreated	83.03	−0.83	1.83	-	-	-	-	0.19	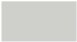
S1	67.92	3.23	52.64	−15.1	4.06	50.8	53.16	5.02	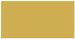
S2	71.10	−5.51	41.84	−11.9	−4.69	40.01	42.01	5.33	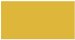
S3	71.00	1.02	65.49	−12.3	1.85	63.65	64.81	3.87	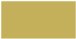
S4	77.66	−4.26	55.05	−5.37	−3.43	53.21	53.59	3.25	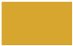
S5	75.21	−0.81	81.94	−7.82	0.02	80.1	80.49	2.99	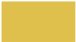
S6	72.95	−0.45	64.78	−10.7	0.37	62.94	63.74	3.05	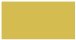
S7	65.54	−0.19	57.76	−17.9	0.64	55.93	58.6	3.62	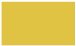
S8	78.89	−5.88	66.1	−4.13	−5.05	64.27	64.6	2.34	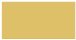
S9	78.97	−5.41	56.63	−4.06	−4.58	54.8	55.14	5.2	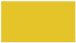
S10	71.13	−2.08	46.2	−11.9	−1.25	44.37	45.95	16.95	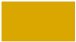
S11	74.95	−2.26	58.42	−8.07	−1.44	56.59	57.18	9.85	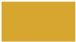
S12	75	−0.31	73.75	−8.02	0.52	71.91	72.36	7.62	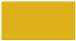
S13	77.2	−3.53	66.86	−5.83	−2.71	65.03	65.35	10.44	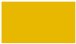
S14	73.18	2.94	76.62	−9.85	3.76	74.79	75.53	6.09	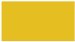
S15	78.94	−4.04	64.09	−4.09	−3.22	62.25	62.47	13.31	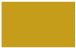
S16	73.68	2.05	81.1	−9.35	2.88	79.27	79.87	4.57	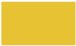
S17	73.85	1.92	84.78	−9.18	2.74	82.95	83.5	6.56	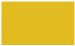
S18	74.23	−0.19	63.23	−8.80	0.64	61.40	62.03	8.74	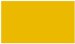
S19	69.14	2.82	56.18	−13.89	3.65	54.35	56.22	8.36	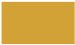
S20	77.48	−5.34	52.91	−5.55	−4.51	51.07	51.57	11.09	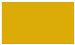
S21	64.48	4.02	66.56	−18.55	4.85	64.73	67.51	14.58	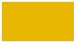
S22	75.75	−5.65	51.22	−7.28	−4.82	49.39	50.15	31.03	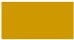
S23	78.51	−5.84	58.94	−4.51	−5.01	57.1	57.5	16.64	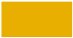
S24	76.33	−0.96	73.76	−6.70	−0.13	71.93	72.24	19.81	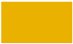
S25	76.07	−2.7	65.8	−6.96	−1.87	63.97	64.37	8.96	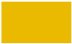
S26	77.76	−2.72	43.41	−5.27	−1.89	41.57	41.95	24.97	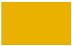
S27	76.25	−0.32	73.44	−6.78	0.51	71.61	71.93	3.31	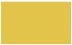

**Table 3:** FTIR peak assignments of undyed and turmeric-extract-dyed nylon 6 samples S3, S11, S20 and S26.

Sample	Peak Position (cm^−1^)	Functional Group	Interpretation
Untreated nylon 6	3298	N–H (amide)	Characteristic amide group of nylon 6
	2924	C–H	Polymer backbone of nylon 6
	1635	Amide I (C=O)	Typical amide linkage of nylon 6
S20	3300	O–H	Broadening indicates adsorption of curcumin and hydrogen bonding
S11	3200–3500	O–H	Presence of curcumin molecules on fiber surface
	1198	C–O/C–N	Characteristic functional groups of curcumin adsorbed onto nylon 6
S26	3200–3500	O–H	Strong hydrogen bonding between curcumin and nylon 6
S3	3200–3500	O–H	Evidence of turmeric dye adsorption
S11–S26	1600–1635	Aromatic C=C/Amide I	Interaction between dye molecules and nylon 6 chains

**Table 4:** Antibacterial assessment of the undyed and dyed nylon samples according to AATCC TM 100 (Quantitative Testing Protocol) against *S. aureus* (ATCC 25923) and *E. coli* (ATCC 25922).

Sample	*S. aureus* ATCC 25923			*E. coli* ATCC 25922		
	CFUs at t_o_	CFUs at t_1_	Reduction %	CFUs at t_o_	CFUs at t_1_	Reduction %
Control	1.54 × 10^5^	1.54 × 10^5^	---	1.85 × 10^5^	1.85 × 10^5^	---
S3	1.54 × 10^5^	2.65 × 10^4^	82.79	1.85 × 10^5^	4.71 × 10^4^	74.54
S11	1.54 × 10^5^	2.01 × 10^4^	86.94	1.85 × 10^5^	4.63 × 10^4^	74.97
S20	1.54 × 10^5^	1.97 × 10^4^	87.20	1.85 × 10^5^	3.87 × 10^4^	79.08
S26	1.54 × 10^5^	1.93 × 10^4^	87.46	1.85 × 10^5^	4.12 × 10^4^	77.72

**Table 5:** The assessment of antioxidant activities of dyed (turmeric extract) and undyed nylon 6 samples in an in vitro antioxidant assay using DPPH^●^ radical decay.

Sample	Absorbance (517 nm)	Antioxidant Activity (%)
Undyed Nylon	2.3211	5.64 ± 1.03
S3	0.74345	68.10 ± 2.37
S11	0.5764	75.15 ± 3.11
S20	0.6648	71.56 ± 2.94
S26	0.6942	70.25 ± 3.29

**Table 6:** Colorfastness results of IR-assisted dyeing of nylon 6 fabric recorded according to the respective testing protocols.

Sample Number	Dry Rubbing (1–5) (ISO 105-X12)	Wet Rubbing (1–5) (ISO 105-X12)	Wash Fastness (1–5) (ISO 105-C06)	Light Fastness (1–8) (ISO 105-B02)
S1	4/5	4/5	4/5	6
S2	4/5	4/5	4/5	6
S3	4/5	4/5	4/5	5
S4	4/5	4/5	4/5	6
S5	4/5	4/5	4/5	6
S6	4/5	4/5	4/5	6
S7	4/5	4/5	4/5	5
S8	4/5	4/5	4/5	6
S9	4/5	4/5	4/5	6
S10	4/5	4/5	4/5	6
S11	4/5	4/5	4/5	5
S12	4/5	4/5	4/5	5
S13	4/5	4/5	4/5	6
S14	4/5	4/5	4/5	6
S15	4/5	4/5	4/5	6
S16	4/5	4/5	4/5	5
S17	4/5	4/5	4/5	5
S18	4/5	4/5	4/5	6
S19	4/5	4/5	4/5	5
S20	4/5	4/5	4/5	5
S21	4/5	4/5	4/5	6
S22	4/5	4/5	4/5	5
S23	4/5	4/5	4/5	6
S24	4/5	4/5	4/5	6
S25	4/5	4/5	4/5	5
S26	4/5	4/5	4/5	5
S27	4/5	4/5	4/5	6

**Table 7:** Three-way factorial ANOVA showing the effects of liquor ratio, temperature, treatment time, and their interactions on K/S values. The analysis was based on 81 observations (27 treatment combinations × 3 independent replicate dyeing processes). The residual degrees of freedom are 81 − 27 = 54. SS = sum of squares; df = degrees of freedom. All factors and interactions were significant at *p* < 0.001.

Source of Variation	Sum of Squares (SS)	df	F-Value	*p*-Value
Liquor ratio	1727.911	2	835.497	<0.001
Temperature	267.653	2	129.418	<0.001
Time	91.058	2	44.029	<0.001
Liquor ratio × Temperature	487.959	4	117.971	<0.001
Liquor ratio × Time	102.256	4	24.722	<0.001
Temperature × Time	591.584	4	143.024	<0.001
Liquor ratio × Temperature × Time	684.119	8	82.698	<0.001
Residual (Error)	55.839	54	—	—

**Table 8:** Minitab-developed (version 22.5.2) design of experiment for the dyeing of nylon fabrics using turmeric extract via the exhaust IR-assisted exhaust dyeing method.

Samples	Liquor Ratio	Temperature °C	Time (Minutes)
S1	1:10	110	60
S2	1:10	90	120
S3	1:10	90	60
S4	1:10	110	90
S5	1:10	130	90
S6	1:10	110	120
S7	1:10	130	120
S8	1:10	90	90
S9	1:10	130	60
S10	1:20	110	60
S11	1:20	90	120
S12	1:20	110	120
S13	1:20	110	90
S14	1:20	130	60
S15	1:20	90	60
S16	1:20	130	90
S17	1:20	130	120
S18	1:20	90	90
S19	1:30	90	60
S20	1:30	110	120
S21	1:30	130	90
S22	1:30	110	60
S23	1:30	90	90
S24	1:30	110	90
S25	1:30	130	60
S26	1:30	130	120
S27	1:30	90	120

## Data Availability

The data that support the findings of this study are available from the corresponding author upon reasonable request.
